# Investigation of Unwanted Oscillations of Electrically Modulated Magnetoelectric Cantilever Sensors

**DOI:** 10.3390/s23115012

**Published:** 2023-05-23

**Authors:** Julius Schmalz, Elizaveta Spetzler, Jeffrey McCord, Martina Gerken

**Affiliations:** 1 Integrated Systems and Photonics, Department of Electrical and Information Engineering, Kiel University, Kaiserstraße 2, 24143 Kiel, Germany; 2 Nanoscale Magnetic Materials, Department of Material Science, Kiel University, Kaiserstraße 2, 24143 Kiel, Germany; elgo@tf.uni-kiel.de (E.S.); jmc@tf.uni-kiel.de (J.M.); 3 Kiel Nano, Surface and Interface Science (KiNSIS), Kiel University, Kaiserstraße 2, 24143 Kiel, Germany

**Keywords:** magnetic field sensor, magnetoelectric, FEM, oscillation, magnetic domains, noise

## Abstract

Magnetoelectric thin-film cantilevers consisting of strain-coupled magnetostrictive and piezoelectric layers are promising candidates for magnetic field measurements in biomedical applications. In this study, we investigate magnetoelectric cantilevers that are electrically excited and operated in a special mechanical mode with resonance frequencies above 500 kHz. In this particular mode, the cantilever bends in the short axis, forming a distinctive U-shape and exhibiting high-quality factors and a promising limit of detection of 70pT/Hz^1/2^ at 10 Hz. Despite this U mode, the sensors show a superimposed mechanical oscillation along the long axis. The induced local mechanical strain in the magnetostrictive layer results in magnetic domain activity. Due to this, the mechanical oscillation may cause additional magnetic noise, deteriorating the limit of detection of such sensors. We compare finite element method simulations with measurements of magnetoelectric cantilevers in order to understand the presence of oscillations. From this, we identify strategies for eliminating the external effects that affect sensor operation. Furthermore, we investigate the influence of different design parameters, in particular the cantilever length, material parameters and the type of clamping, on the amplitude of the undesired superimposed oscillations. We propose design guidelines to minimize the unwanted oscillations.

## 1. Introduction

Magnetoelectric (ME) cantilevers have been a topic of significant research in recent years due to their potential biomedical applications. These cantilevers are generally used for both energy harvesting [1,2,3] and magnetic field sensing at room temperature [4,5,6], making them ideal for a wide range of biological applications [7,8,9]. In biomedical research, magnetic field sensing is of great interest, as it can be used to source localization and detect magnetic nanoparticles used in bioassays. Cantilever sensors consist of a magnetostrictive (MS) and a piezoelectric layer (PE) that work together to convert magnetic fields into an induced electric charge. The MS layer changes length due to Joule magnetostriction when a magnetic field is applied, causing the cantilever to deflect. This deformation results in a change in the polarization of the PE layer, leading to a measurable surface charge. By measuring the surface charge, the magnetic field can be quantified, allowing for the detection of small magnetic fields with high sensitivity.

An exciting possibility for the operation of magnetoelectric (ME) sensors is the use of electrically modulated ME sensors, which have potential applications in the field of biomedicine. The converse magnetoelectric effect is utilized in this approach [10,11], where an alternating electric potential is applied to the piezoelectric (PE) layer, exciting the cantilever. The PE layer responds to the change in polarization with mechanical stress due to the inverse piezoelectric effect, resulting in a deflection of the cantilever. As a consequence, the magnetization *M* of the cantilever changes according to the inverse magnetostrictive effect, which can be detected using a surrounding pickup coil [8,12,13]. The application of an external magnetic field leads to an amplitude modulation at the excitation frequency, which can be detected by the induced voltage of the pickup coil.

This modulation scheme allows the measurement of low-frequency magnetic fields, while mitigating the effects of low-frequency noise in the electronics. Moreover, this approach does not limit the measurement to the resonance frequency of the cantilever, making it particularly useful in biomedical applications where the detection of low-frequency magnetic fields is of interest.

**Figure 1:**
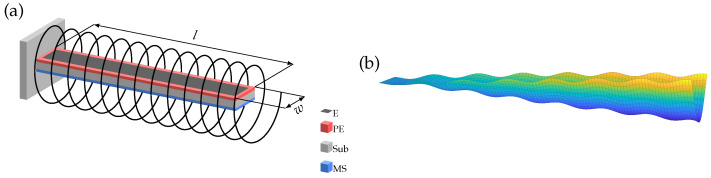
(**a**) Schematic of sensor representation including substrate (Sub), magnetostrictive layer (MS), piezoelectric layer (PE), and top electrode (E) surrounded by the readout coil. (**b**) Displacement of U mode with superimposed oscillation.

In this study, we investigated electrically excited cantilevers operating in a special mechanical mode, where the cantilever is bent in the short axis resulting in a U-shape as depicted in Figure 1b. These U modes have resonance frequencies above 500 kHz for the used geometry, and for readout, the cantilever is surrounded by a pickup coil (Figure 1a).

The U mode has demonstrated superior performance compared to other surrounding mechanical modes, exhibiting the highest displacement and a quality factor of Q≈1000, as well as an excellent limit of detection (LOD) of 70 pT/Hz^1/2^ at 10 Hz [8,12,13]. In addition, this mode exhibits high stresses along the short axis, and therefore, generates a significant magnetic response. This mode also generates a significant magnetic response due to high stresses along the short axis. Moreover, using high frequencies results in less damping and higher quality factors *Q*, as interaction with air molecules is minimized compared to traditional modes at 1 kHz [14]. Another contributing factor to the high-quality factor of the U-mode may be lower mechanical losses in the clamp region in combination with lower magnetic losses due to a more uniform domain structure.

Despite the advantages of this mode, many sensors still exhibit an unwanted mechanical oscillation along the long axis.

Due to the induced local mechanical strain in the magnetostrictive layer, these oscillations cause local effective magnetic anisotropy changes inside the magnetic layer due to the inverse magnetostrictive effect [9,15,16]. The local stress-induced magnetic anisotropy changes may lead to magnetic domain effects (cf. Section 2), which are expected to cause extra magnetic noise contributions and to deteriorate the sensor’s limit of detection (LOD) [17,18]. In general, both high sensitivity and low intrinsic noise are required to achieve a low limit of detection (LOD) [8]. Recent research has shown that optimizing the noise contribution of the magnetostrictive layer by using magnetostrictive multilayers is a key factor in achieving low LODs [17,19]. This concept has been shown to be effective for other ME sensor concepts [20,21]. Reducing the oscillations, which reduces the interference to the magnetostrictive layer, appears to be an appropriate approach to achieving low LODs.

In this paper, we compare finite element method (FEM) simulations with measurements of magnetoelectric cantilevers to investigate the presence of the oscillation in simulation and to eliminate external effects disturbing the measurement. Further, we investigate the influence of different design parameters, such as the length of the cantilever, the material parameters, and the type of clamping, on the amplitude of the undesired superimposed oscillations in the long direction of the cantilever. In this way, we propose design guidelines to minimize the oscillations and also possible magnetic noise contributions.

## 2. Induced Magnetization and Magnetic Domain Response

**Figure 2:**
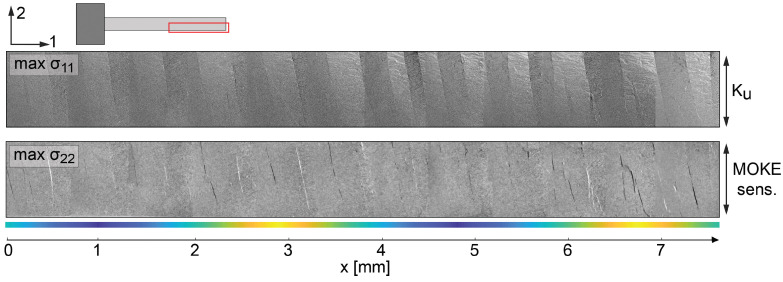
MOKE microscopy image showing the tip of cantilever (red area of inset) excited at 516 kHz with no magnetic bias field applied. The left side of the cantilever is fixed to a printed circuit board. The max stress is shown at $\sigma_{11}$ (↔) and $\sigma_{22}$ stress (↕). The direction of the induced magnetic anisotropy $K_{u}$ and the MOKE sensitivity are shown. Regions with low (blue) and high (yellow) magnetic domain wall activity are indicated.

The principal effects of the mechanical mode activity on the magnetic domain activity are investigated by operando time-resolved magnetooptical Kerr effect (MOKE) microscopy [22,23]. A corresponding MOKE image of a single layer magnetostrictive film (FeCoSiB) with 2 μm thickness of a cantilever [13] excited at f=516 kHz and U=2Vpp without the application of a magnetic bias field is shown in Figure 2. The axis of magnetooptical sensitivity is vertical, as indicated by arrows. The intrinsic induced anisotropy is perpendicular to the cantilever axis. Therefore, the domains and the domain walls are oriented along the short axis of the cantilever. The alternating stripes of different contrasts oriented in the short axis of the cantilever show the spatial domain activity due to the alternating stress acting on the magnetization.

The stress-induced anisotropy in the case of max $\sigma_{11}$ stress (↔ in Figure 2) is oriented perpendicular to the intrinsic magnetic anisotropy axis. Here, the domain walls are immobile, but the magnetization within the domains rotates. The main shape of the U mode can, therefore, be seen in the MOKE micrograph, as the changing contrast of the domains increases towards the center of the cantilever. The domain activity further spatially changes along the long axis of the cantilever. This is a direct consequence of the U mode amplitude variation along the cantilever.

These effects are barely visible at max $\sigma_{22}$ stress, where an increase in stress does not lead to a substantial magnetic response within the magnetic domains, as the domain magnetization is already aligned along the preferred direction as the initial uniaxial anisotropy is oriented parallel to the stress-induced anisotropy. On the other hand, at max $\sigma_{22}$ stress (↕ in Figure 2), the domain walls move. In fact, adjacent domain walls displace in opposite directions, as can be seen from the alternating dark and bright domain wall contrasts in the MOKE micrograph. This indicates that the superimposed oscillations result in magnetic domain (wall) activity. The domain wall induced MOKE contrast for the max $\sigma_{22}$ stress apparently varies along the length of the cantilever, further suggesting a connection between the superimposed oscillations and the domain wall activity. Alternating regions with low and high magnetic domain wall activity are indicated in Figure 2. Note that the overall amplitude of the domain wall activity does not decrease significantly within the field of view as for the domain activity shown for $\sigma_{11}$.

Overall, the MOKE imaging data indicate an influence of the superimposed oscillation on the magnetization response. The domain and domain wall response does not contribute to the ME sensor signal, but is expected to influence the overall noise performance of the sensor [17,20]. Strategies for reducing or eliminating the additional oscillations are derived from modeling, as will be shown next.

## 3. Modeling

### 3.1. Equation System

The coupled mechanical, electric and magnetic behavior of the magnetoelectric cantilevers were modeled by the coupled differential equation system consisting of Newton’s law and Maxwell’s Equations [24,25]:

(1) $$\begin{array}{cc} \nabla \;\cdot \;T & =-\rho \omega^{2}\vec{u} \end{array}$$

(2) $$\begin{array}{cc} \nabla \;\cdot \;\vec{D} & =0 \end{array}$$

(3) $$\begin{array}{cc} \nabla \;\cdot \;\vec{B} & =0. \end{array}$$

where ***T*** is the stress tensor, ρ the mass density, ω the angular frequency, $\vec{u}$ the displacement vector, and $\vec{D}$ and $\vec{B}$ the electric and magnetic flux density, respectively. Here, it is assumed that space charges and conduction currents are negligible.

A linearized set of constitutive material equations are evaluated at the small-signal operating point:

(4) $$\begin{array}{cc} T & =c^{EH}S-e_{e}\vec{E}-e_{m}^{T}\vec{H} \end{array}$$

(5) $$\begin{array}{cc} \vec{D} & =e_{e}S+\varepsilon \vec{E} \end{array}$$

(6) $$\begin{array}{cc} \vec{B} & =e_{m}S+\mu \vec{H} \end{array}$$

where S describes the strain tensor, $c^{\mathrm{EH}}$ the stiffness tensor, $e_{e}\;\mathrm{and}\;e_{m}$ the strain to field coupling constants, $\vec{E}\;\mathrm{and}\;\vec{H}$ the electric and magnetic field, respectively, and ε and μ the permittivity and permeability, respectively.

The elasticity relation is given by Equation (7) and scalar potentials are assumed for the electric and the magnetic fields (Equations (8) and (9), respectively):

(7) $$\begin{array}{cc} S & =1/2\left[{\left(\nabla \vec{u}\right)}^{T}+\nabla \vec{u}\right] \end{array}$$

(8) $$\begin{array}{cc} \vec{E} & =-\nabla V \end{array}$$

(9) $$\begin{array}{cc} \vec{H} & =-\nabla V_{m}. \end{array}$$

where *V* is the electric potential and $V_{m}$ is the magnetic potential. A 3D simulation of the ME sensor is performed using COMSOL Multiphysics 6.1^®^, implementing the above linear equations in the general form partial differential Equation (PDE) interface as a small-signal approximation.

The used material parameters are given in Appendix A.

### 3.2. Simulation Approach

The modeling was performed in two different manners. For the pure mechanic simulation ($\vec{E}=0$, $\vec{H}=0$), a simple eigenvalue study is carried out with a given start frequency for the desired U mode. The desired U mode is manually selected from a set of eigenmodes above a certain start frequency. Especially for the more distorted eigenmodes, the choice is not always obvious.

**Figure 3:**
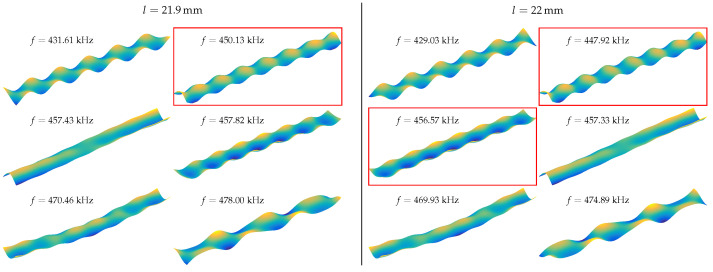
Displacement field for different modes for l=21.9 mm (**left**) and l=22 mm (**right**) cantilever lengths. The possible U mode selections are highlighted with a red rectangle.

An example of this can be seen in Figure 3: for a length of 21.9 mm, there is one possible U mode at f=450.13 kHz. However, for a length of 22 mm, there are two possible modes at f=447.92 kHz and f=456.57 kHz. The U mode frequency decreased with increasing cantilever length. Consequently, the mode at f=447.92 kHz could be selected. Nevertheless, the next clean U mode has a frequency of around f=455 kHz. Due to this frequency jump, the mode with the higher frequency can also be selected for a length of 22 mm. These frequency jumps make the identification of the more distorted U modes not straightforward. Therefore, the eigenvalue study searches for a given number of modes and the U modes are selected manually for further processing.

In order to analyze the magnetic response of a sensor due to electric excitation, a more complex approach is required, as the eigenfrequency study does not consider the electrical excitation of the cantilever, which induces additional stress, and therefore, changes the resonance frequency slightly. Therefore, a two-stage approach is conducted, consisting of an eigenfrequency study as a preliminary step to estimate the resonance frequency followed by a frequency domain study to determine the resonance frequency precisely in the presence of an electric excitation. Skipping the eigenfrequency study and performing only the frequency domain study would tremendously increase the simulation time, as one would have to expand the frequency range to determine the resonant frequency without a starting point.

Additionally, the equations describing the magnetostriction (Equations (4) and (6)) have to be disabled for the eigenfrequency study due to numerical instabilities. Overall, the frequency domain study is a necessary second step to obtain the magnetic response of the sensor.

## 4. Origin of Superimposed Oscillations

### 4.1. U Mode in the Experiment and Simulation

**Figure 4:**
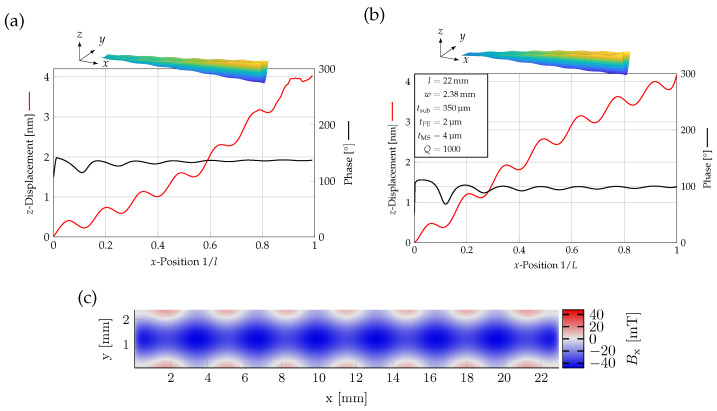
(**a**) High-speed vibrometer measurement displaying the presence of a superimposed oscillation at f=516kHz. Center-line measurement for a 22 mm long and 2.38 mm wide cantilever showing the displacement (red) and phase (black). (Raw data provided by Hayes et al. [13]). (**b**) Displacement (red) and phase (black) along the center line from FEM. f=512kHz. The insets show 3D displacement plots for the measured and calculated cantilever response (**c**). Change of magnetization $B_{x}$ of a free–free cantilever showing opposite field direction regions at the outer edges. These lead to a reduced total magnetic field due to the superimposed oscillations.

During the electrical excitation of the cantilever, the presence of a superimposed oscillation was observed by measuring the mechanical displacement of the cantilever using a high-speed vibrometer [13], as illustrated in Figure 4a. To confirm that this effect was not simply a measurement artifact, a finite element method (FEM) simulation was conducted using identical dimensions and materials to those employed in the experiment (Figure 4b). The model described in the previous Section 3, which has been validated in the past for comparable setups in both fundamental and higher order modes, was used for the simulation [25,26,27,28].

Both measurement and simulation results show an increasing displacement with increasing *x*-position and with an obvious oscillation. The relative amplitude of the oscillation was slightly larger for the FEM. Due to the electric excitation of the cantilever, there is an oscillation of the phase in both the measurement and simulation results. This is likely due to the fact that the electrodes were not optimized for U-shaped excitation, and thus, interfered with the mechanical resonance mode.

The oscillations are causing a local strain in the cantilever, which generates a local magnetic flux due to the inverse magnetostriction effect, as seen in Figure 4c. Here, the *x*-component of the magnetic flux was plotted in the xy-plane of the cantilever in a free–free configuration. The magnetic flux oscillates in the same pattern as the displacement. These local effects are expected to influence the domain structure of the magnetostrictive layer (see Figure 2), likely causing additional magnetic noise.

### 4.2. Bending Modes

As the oscillations occur both in the experiment and in the simulation, the question of their origin arises. For this purpose, a cantilever with a length of 22 mm and a width of 2.38 mm, chosen according to the experimental setup, is considered.

The oscillations could be caused by superposition with a higher order bending mode. However, the frequency of a 15th order bending mode at f=575 kHz is much higher than the current resonance frequency of f=473 kHz. Therefore, the origin cannot be a superposition with a bending mode.

### 4.3. Longitudinal Modes

An alternative explanation could be that the superimposed oscillations are caused by longitudinal modes.

**Figure 5:**
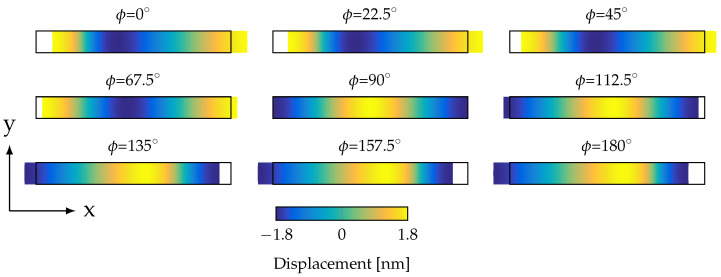
Displacement of a cantilever in the longitudinal mode closest to the U mode at f=349 kHz in a top view for different phase angles ϕ. The colored area shows the deformation illustrating the movement from the neutral position (depicted by the black rectangle).

To investigate this in more detail, a free–free cantilever was used. The origin of the superimposed oscillation was narrowed down to a pure mechanical phenomenon by reducing the complexity of the model to a simple silicon cantilever without any active layers. For a longitudinal mode, there has to be movement in the long- or *x*-direction of the cantilever, as illustrated in Figure 5. Nevertheless, there was no longitudinal movement for the U modes, which also rules out a superimposed longitudinal mode.

### 4.4. Influence of Q-Factor

For modes with a low-quality factor, there can be modal overlap between two or more modes due to their wider bandwidth causing multiple modes to superimpose to a hybrid mode. To eliminate this effect as the source of the oscillations, the simulations were also performed with a very high-quality factor (*Q*). However, even with a *Q* = 100,000, there is no change in the mode displacement, while solely the imaginary part of the eigenfrequency was reduced.

### 4.5. Influence of Poisson’s Ratio

**Figure 6:**
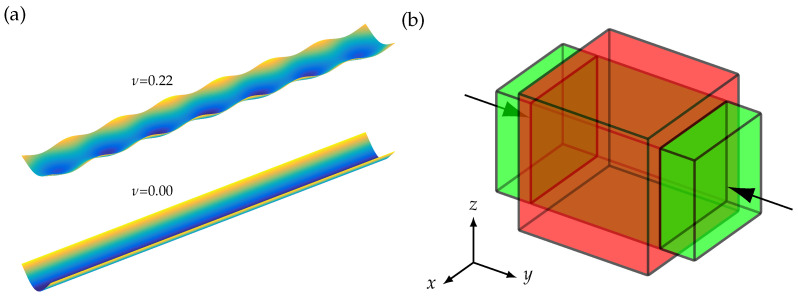
(**a**) Displacement of 22 mm long and 2.45 mm wide cantilever at 447 kHz with oscillations for ν=0.22 and no oscillations for ν=0. (**b**) According to the Poisson’s ratio, an axial strain in the y-direction (black arrows) causes a transverse strain in the *x*- and *z*-direction. When the length of the green cuboid is compressed, the width is expanded to the red cuboid.

Furthermore, there is a very basic principle left, which can cause the undesired deformation: when the cantilever is bending in the U mode, there is also a transverse contraction in the long cantilever axis due to the material properties. As illustrated in Figure 6b, when there is axial strain in *y*-direction, there is also transverse strain in the *x*-direction and the ratio of the transverse strain to the axial strain is defined as the Poisson’s ratio of a material.

For the cantilever operated in the U mode, this means that the transverse contraction might cause the superimposed oscillation in the long axis of the cantilever.

This assumption was analyzed by modifying the Poisson’s ratio ν of the substrate to be zero, resulting in a clean U mode bending without any superimposed oscillations. The superimposed oscillation cannot be entirely eliminated for the magnetoelectric cantilever, as silicon has a ν=0.22.

## 5. Strategies to Reduce Oscillations

In Section 4 the presence of the superimposed oscillation in the long cantilever axis were shown and the Poisson’s ratio was identified as the source of the oscillations. In this section, other parameters influencing the strength of the oscillations will be discussed. These parameters are the length and the type of clamping of the cantilever.

### 5.1. Cantilever Length

As the resonance frequency of the U shape in the short axis is mainly given by the cantilever width, a change in the cantilever length does not influence the U mode frequency significantly. This is advantageous for the comparison of the results by keeping as many parameters constant as possible.

**Figure 7:**
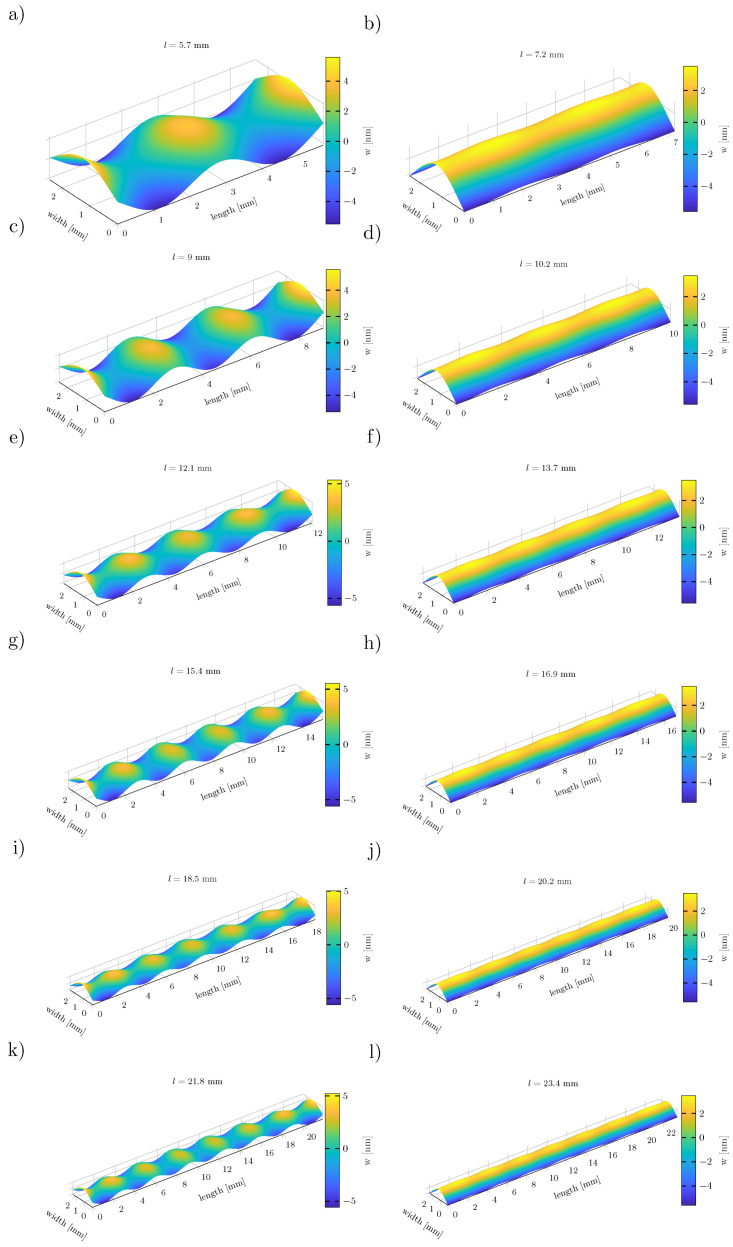
Displacement of silicon cantilevers with different oscillation amplitudes. On the left, the cantilevers have maximum amplitudes for lengths of (**a**) l=5.7 mm, (**c**) l=9.0 mm, (**e**) l=12.1 mm, (**g**) l=15.4 mm, (**i**) l=18.5 mm, (**k**) l=21.8 mm. On the right, the cantilevers have minimum amplitudes for lengths of (**b**) l=7.2 mm, (**d**) l=10.2 mm, (**f**) l=13.7 mm, (**h**) l=16.9 mm, (**j**) l=20.2 mm, (**l**) l=23.4 mm. All cantilevers have a fixed width of 2.45 mm and a thickness of 350 μm.

Figure 7 shows the displacements of cantilevers, where the lengths were chosen to have minimal (right) and maximal (left) oscillations. The cantilevers on the left have significantly fewer oscillations than the ones on the right.

**Figure 8:**
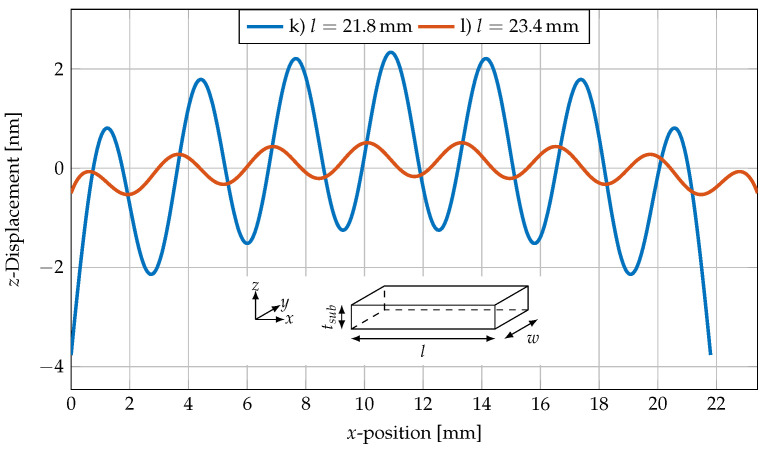
*z*-Displacement field of a silicon cantilever with a fixed length of 21.8mm (corresponding to subfigure (k) in Figure 7) and 23.4 mm (corresponding to subfigure (l) in Figure 7), a width of 2.45 mm, and thickness of 350 μm. The *z*-displacement is plotted along the middle of the top surface of the cantilever as a function of *x*.

The strength of the oscillation is highly dependent on the length of the cantilever. In Figure 8, the displacement is exemplarily shown for 21.8 mm and 23.4 mm long cantilevers with a width of 2.45 mm and thickness of 350 μm, corresponding to Figure 7k,l. The active MS and PE layer are omitted to reduce the complexity of the multiphysics model to a pure mechanical model. By this, the influence of the active layer on the oscillation was eliminated.

The amplitude of the oscillation is approximately 1.77 nm for Figure 7k and 0.34 nm for Figure 7l. The imaginary zero line of the oscillation is slightly bent.

**Figure 9:**
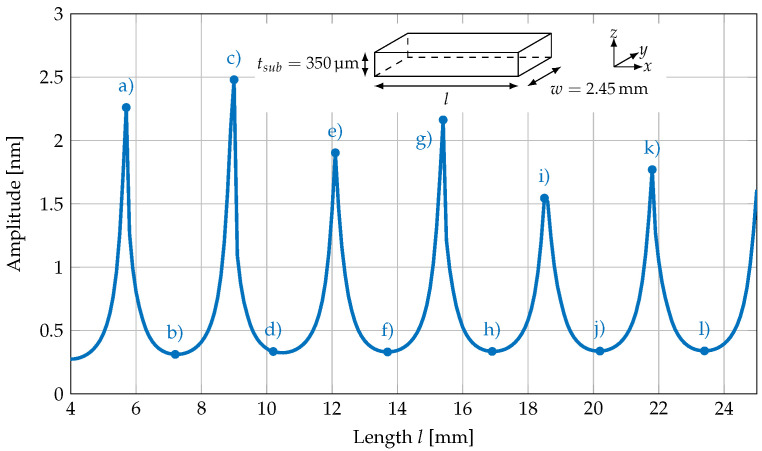
Oscillation amplitude of a silicon cantilever with a fixed width of 2.45 mm and thickness of 350 μm for lengths of 4–25 mm. The labels (a–l) in the figure correspond to the three-dimensional displacement plots of the cantilevers shown in Figure 7.

The amplitude was determined applying a sine fit and used in Figure 9, where the amplitude of the oscillation for lengths of 4–25 mm is plotted for a cantilever with a fixed width of 2.45 mm and a silicon substrate thickness of 350 μm.

The graph in Figure 9 shows repetitive maximal and minimal oscillation amplitudes for different cantilever lengths. The periodicity is 3.25 mm, which also matches with the wavelength of the oscillations along the cantilever length in Figure 8. It is also in agreement with the results of the MOKE imaging analysis shown in Figure 2.

### 5.2. Full Cantilever Stack

The results of a silicon cantilever showed that superimposed oscillations are a pure mechanical effect, which can only be minimized and not fully eliminated. In order to establish a design guide for magnetoelectric cantilevers operated in the U mode, the fully operational cantilevers with active layers of piezoelectric and magnetostrictive materials were investigated.

**Figure 10:**
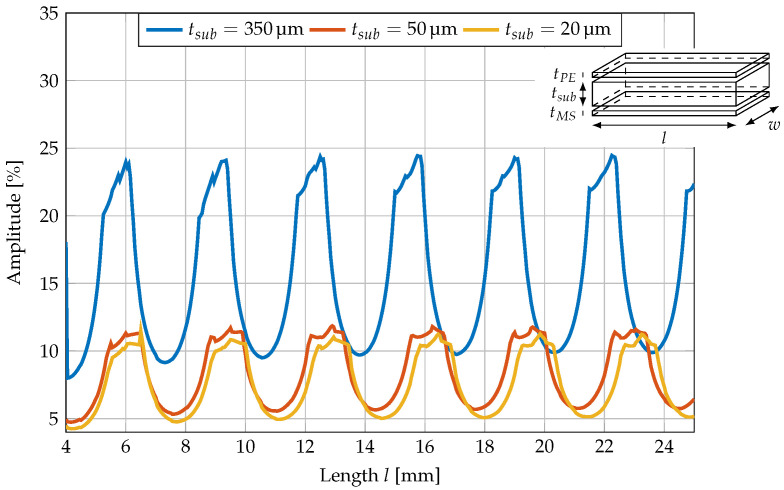
Relative displacement amplitude of superimposed oscillation to displacement of U mode for a silicon cantilever of different lengths and substrate thicknesses with a width of 2.45 mm. The relative *z*-displacement is plotted along the middle of the top surface of the cantilever as a function of *x*. There is no significant influence of the substrate thickness on the periodicity of the superimposed oscillation.

The relative displacement amplitudes of the superimposed oscillation for a silicon cantilever with different lengths and a width of 2.45 mm are shown in Figure 10. To further investigate the influence of the substrate thickness on the oscillation amplitude, three different substrate thicknesses were simulated. For better visibility, the amplitude of the superimposed oscillations is scaled to the amplitude of the U mode.

Compared to the results without the active layers, the periodicity of the oscillation is the same with 3.25 mm for a 350 μm thick substrate. For 50 μm, it is 3.4 mm and for 20 μm, 3.45 mm. Thus, the periodicity is only slightly decreasing with increasing substrate thickness.

Compared to the results for a pure silicon cantilever, the width of the maxima was increased for the full cantilever stack. Furthermore, the oscillation amplitude was reduced with decreasing substrate thickness.

**Figure 11:**
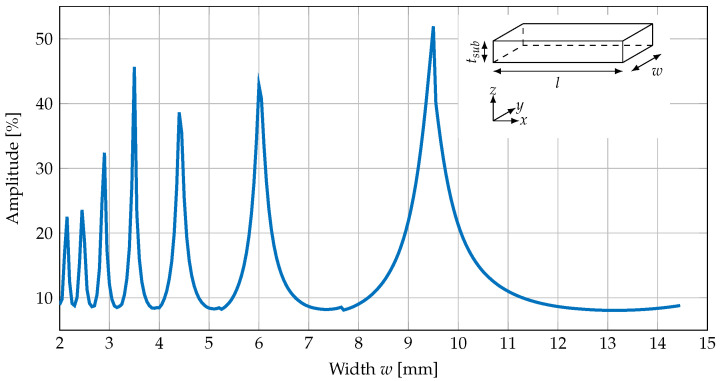
Relative displacement amplitude of superimposed oscillation to displacement of U mode for a silicon cantilever of different widths of 2–15 mm with a length of 22 mm and a substrate thickness of 350 μm.

However, the periodicity along the long axis of the cantilever is increasing with the width of the cantilever as shown in Figure 11. Here, the relative displacement is shown for a 22 mm long cantilever with a 350 μm thick substrate and 4 μm thick PE and MS layers.

**Figure 12:**
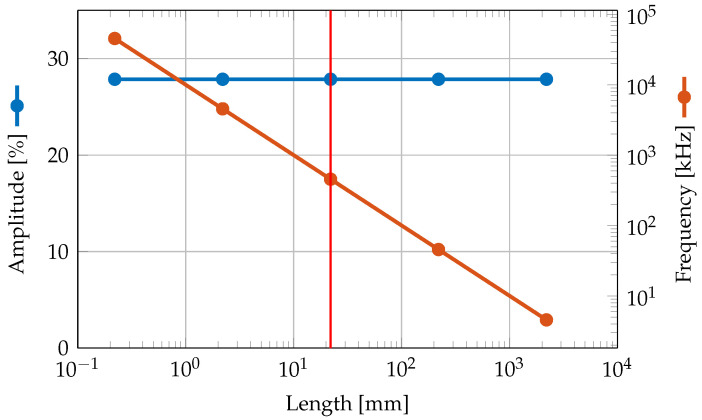
Relative displacement amplitude of superimposed oscillation to displacement of U mode for a silicon cantilever with lengths between 220 μm and 2.2 m. The cantilever with a length of 22 mm, a width of 2.45 mm and a substrate thickness of 350 μm is marked with a red line. For the remaining data points, the cantilever was scaled according to the given length.

Changing the length, width, and height of the cantilever at the same time, the frequency decreases with 1/x, where *x* is the scaling factor. However, this has no effect on the strength of the oscillations as shown in Figure 12 due to the linearity of the model. For measurements of real cantilevers, scaling is expected to have an effect as material parameters change due to edge effects becoming more significant.

Overall, the length-sweep data for magnetoelectric cantilevers show that the active layers are not the cause of the oscillation, but have a minimal effect on it. However, the width of the cantilever has a distinct influence on the periodicity. Altogether, there are certain length regions with minimal oscillation, which are suitable for magnetoelectric measurements.

### 5.3. Position of the Clamping

**Figure 13:**
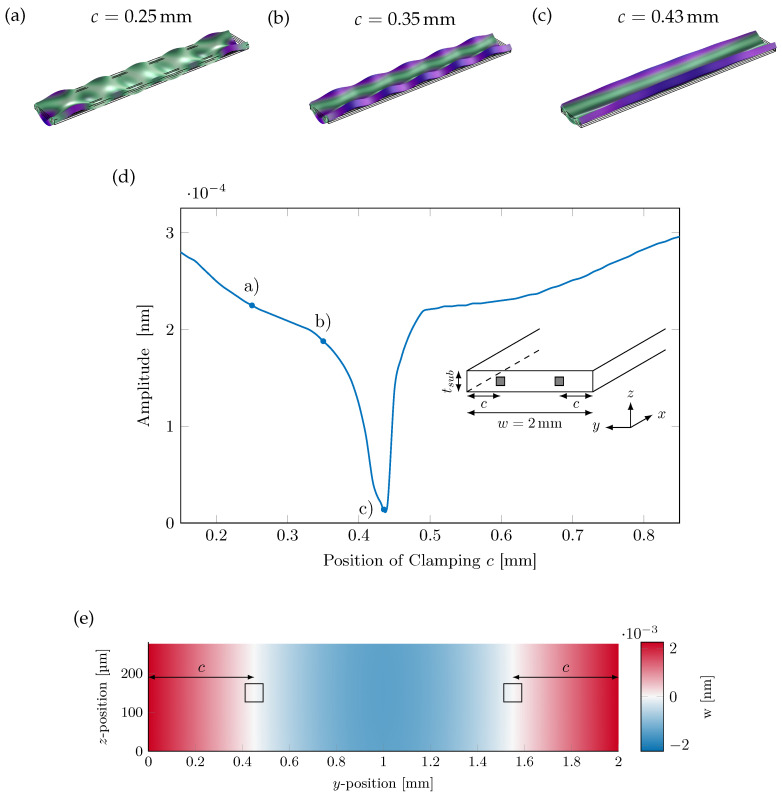
Oscillation amplitude for different clamping positions for a fixed-fixed cantilever consisting of a 14 × 2 × 0.3 mm^3^ silicon substrate and 2 μm thick MS and PE layers. (**a**–**c**) A 3D view of the displacement field is shown for c=0.25mm, c=0.35mm, and c=0.43mm. (**d**) shows a graph of the oscillation amplitude for different clamping positions *c* with a minimum for c=0.43mm. (**e**) *z*-Displacement in the yz-plane at the end (x=0mm) of a free–free cantilever with a length of 14 mm, a width of 2 mm, a substrate thickness of 300 μm, a piezoelectric layer thickness of 2 μm, and a magnetostrictive layer thickness of 4 μm. There are two positions of minimal displacement at ≈0.43 mm and 2 mm − 0.43 mm ≈ 1.57 mm.

The strength of the oscillation is not only dependent on the length or Poisson’s ratio, but also on other factors, e.g., the type of clamping of the cantilever. The previous results were obtained for a free–free cantilever with no clamping. In an experiment, the cantilever has to be fixed in a certain fashion. This can be done by clamping one side of the cantilever, as shown in Figure 8, resulting in a fixed-free cantilever. To allow the cantilever to be free on both sides, it has to be clamped at certain points of least movement, as shown in the inset in Figure 13d by the dark grey square with a size of 100 μm. These clamping points are located at both sides of the cantilevers’ long axis. Here, the exact positions of these points are investigated.

In Figure 13a–c, the displacements with different strengths of oscillation depending on the position *c* of the clamping are shown. The oscillation is the strongest where the cantilever (a) is clamped at 0.25 mm from the outer edge, (b) is clamped at c=0.35 mm with slightly reduced oscillations, and (c) has minimal oscillations at c=0.43 mm. In (d), the amplitude of the oscillation for the position of the clamping of 0.1–0.9 mm is shown. The minimum is at c=0.43 mm and corresponds to the position of the least displacement of the yz-face (Figure 13e) of a free–free cantilever. Due to the high slope, small changes in the position cause large oscillation amplitudes. As a result of the clamping, the displacement is reduced by three orders of magnitude compared to a free–free cantilever in the simulation (Figure 8).

## 6. Discussion and Conclusions

The present study has verified the superimposed mechanical oscillation observed in vibrometer measurements of ME sensors by FEM simulations and identified the transverse contraction of the material as the origin of the oscillation.

Hence, the superimposed oscillations are not caused by a multiphysical effect, but a pure mechanical effect, which was confirmed by elimination of the transverse contraction (ν=0) in simulation; while the Poisson’s ratio of the material cannot be eliminated entirely, selecting a material with a smaller Poisson’s ratio should reduce the oscillation.

Experiments were carried out to analyze the effect of local field changes on the magnetic domain activity of cantilevers (Figure 2), which indicated an influence of the superimposed oscillations on the magnetic response. Similar results were obtained using FEM simulations, which showed that oscillations have a significant effect on the magnetization of the cantilever, leading to a correlation between experiments and simulations. The domain wall response is expected to influence the overall noise performance of the sensor, as discussed in Section 2. The oscillations are also expected to contribute to the noise level around the carrier frequency, which occurs due to the upconversion of low-frequency noise and the periodic magnetization processes in the magnetic phase [14]. In this respect, minimizing the oscillation is recommended to reduce the unwanted magnetic oscillation and improve the sensor performance.

For this purpose, several approaches have been investigated: the oscillations can be reduced by changing the length or width of the sensor to specific values or by reducing the substrate thickness, and in the case of a clamped cantilever, by optimizing the type of clamping.

In terms of cantilever dimensions, for example, a 60% reduction in amplitude can be achieved by selecting an appropriate length for the cantilever under investigation. A further 50% reduction in oscillations can be achieved by reducing the substrate thickness of the cantilever. By changing the width of the bending cantilever, a reduction of approximately 85% can be achieved for wide cantilevers. Using the simulation results, it is possible to select the length/width combinations where oscillations are minimized. On the other hand, scaling the overall dimensions changes the frequency of the U-mode, but does not change the presence of oscillations due to the linearity of the model.

To sum up, this study proposes a design guide to minimize the strength of the oscillations by selecting the length/width combination of the cantilever accordingly. However, it should be noted that the parameter length and width sweep needs to be performed for each change in the remaining parameter setup of the cantilever, such as the thickness of the layers. Additionally, we suggest future research topics, such as noise measurements, to verify the effect of the oscillations. Fabrication of sensors with defined dimensions would be required to have a sensor with maximum and minimum oscillations, which would be of high interest for further investigation.

Overall, our results provide valuable insights into the origin of the superimposed oscillations and offer useful guidelines for reducing the oscillations and improving the performance of ME sensors and further research can continue to optimize sensor performance.

## Data Availability

The data that support the findings of this study are available from the corresponding author upon reasonable request.

## Appendix A. Material Parameters

Silicon [29]:

$$\begin{array}{cc} c_{\mathrm{Si}}^{\mathrm{EH}} & =\left(\begin{array}{cccccc} 216 & 84 & 84 & 0 & 0 & 0\\ 84 & 216 & 84 & 0 & 0 & 0\\ 84 & 84 & 216 & 0 & 0 & 0\\ 0 & 0 & 0 & 66 & 0 & 0\\ 0 & 0 & 0 & 0 & 66 & 0\\ 0 & 0 & 0 & 0 & 0 & 66 \end{array}\right)\;\mathrm{GPa},\\ \rho_{\mathrm{Si}} & =2329\;kg/m^{3},\\ \varepsilon_{\mathrm{Si}} & =\left(\begin{array}{ccc} 107 & 0 & 0\\ 0 & 107 & 0\\ 0 & 0 & 107 \end{array}\right)\;pF/m,\\ \mu_{\mathrm{Si}} & =\left(\begin{array}{ccc} 0.4\pi & 0 & 0\\ 0 & 0.4\pi & 0\\ 0 & 0 & 0.4\pi \end{array}\right)\;\mu H/m. \end{array}$$

AlN [29]:

$$\begin{array}{cc} c_{\mathrm{AlN}}^{\mathrm{EH}} & =\left(\begin{array}{cccccc} 410 & 149 & 99 & 0 & 0 & 0\\ 149 & 410 & 99 & 0 & 0 & 0\\ 99 & 99 & 389 & 0 & 0 & 0\\ 0 & 0 & 0 & 125 & 0 & 0\\ 0 & 0 & 0 & 0 & 125 & 0\\ 0 & 0 & 0 & 0 & 0 & 125 \end{array}\right)\;\mathrm{GPa},\\ \rho_{\mathrm{AlN}} & =3268\;kg/m^{3}\\ \tan\delta_{\mathrm{AlN}} & =0.001,\\ e_{e,\mathrm{AlN}} & =\left(\begin{array}{cccccc} 0 & 0 & 0 & 0 & -0.48 & 0\\ 0 & 0 & 0 & -0.48 & 0 & 0\\ -0.58 & -0.58 & 1.55 & 0 & 0 & 0 \end{array}\right)\;N/Vm,\\ \varepsilon_{\mathrm{AlN}} & =\left(\begin{array}{ccc} 80 & 0 & 0\\ 0 & 80 & 0\\ 0 & 0 & 80 \end{array}\right)\;pF/m,\\ \mu_{\mathrm{AlN}} & =\left(\begin{array}{ccc} 0.4\pi & 0 & 0\\ 0 & 0.4\pi & 0\\ 0 & 0 & 0.4\pi \end{array}\right)\;\mu H/m. \end{array}$$

FeCoSiB [29]:

$$\begin{array}{cc} c_{\mathrm{FeCoSiB}}^{\mathrm{EH}} & =\left(\begin{array}{cccccc} 150 & 45 & 45 & 0 & 0 & 0\\ 45 & 150 & 45 & 0 & 0 & 0\\ 45 & 45 & 150 & 0 & 0 & 0\\ 0 & 0 & 0 & 40 & 0 & 0\\ 0 & 0 & 0 & 0 & 40 & 0\\ 0 & 0 & 0 & 0 & 0 & 40 \end{array}\right)\;\mathrm{GPa},\\ \rho_{\mathrm{FeCoSiB}} & =7250\;kg/m^{3},\\ \varepsilon_{\mathrm{FeCoSiB}} & =\left(\begin{array}{ccc} 8854 & 0 & 0\\ 0 & 8854 & 0\\ 0 & 0 & 8854 \end{array}\right)\;pF/m,\\ e_{m},\mathrm{FeCoSiB} & =\left(\begin{array}{cccccc} 8500 & -2833.3 & -2833.3 & 0 & 0 & 0\\ 0 & 0 & 0 & 0 & 0 & 0\\ 0 & 0 & 0 & 0 & 0 & 0 \end{array}\right)\;N/Am,\\ \mu_{\mathrm{FeCoSiB}} & =\left(\begin{array}{ccc} 1131 & 0 & 0\\ 0 & 1131 & 0\\ 0 & 0 & 1131 \end{array}\right)\;\mu H/m. \end{array}$$
